# Cardiac manifestations in adult MELAS syndrome (mitochondrial encephalomyopathy with lactic acidosis and stroke-like episodes syndrome)– a cross-sectional study

**DOI:** 10.1186/s13023-025-03534-5

**Published:** 2025-02-10

**Authors:** Dietrich Stoevesandt, Axel Schlitt, Philipp Röntgen, Torsten Kraya

**Affiliations:** 1 Department of Radiology, University Hospital Halle-Saale, Ernst-Grube Str. 40, 06097 Halle/Saale, Germany; 2 Medical Faculty, Martin Luther-University Halle-Wittenberg, Halle/Saale, Germany; 3 Paracelsus-Harz-Clinic Bad Suderode, Quedlinburg, Germany; 4 Department of Neurology, University Hospital Halle-Saale, Ernst-Grube Str. 40, 06097 Halle/Saale, Germany; 5 Department of Neurology, St Georg Hospital Leipzig, Ernst-Grube Str. 40, 06097 Halle/Saale, Leipzig, Germany

**Keywords:** MELAS, Cardiac disease, Cardiac MRI, Cardiomyopathy, M.3243A > G mutation

## Abstract

**Backround:**

Cardiac involvement has been reported in different mitochondrial geno- and phenotypes, including mitochondrial myopathy, encephalopathy, lactic acidosis and stroke-like (MELAS) syndrome. However, cardiac manifestations are diverse and not well described.

**Methods:**

We prospectively examined cardiac manifestations in 11 adult patients with MELAS syndrome harboring the MTTL1 m.3243A < G-mutation using patient records, cardiac MRI (1.5 Tesla), echocardiography, electrocardiogram (ECG), laboratory tests of cardiac markers (CK, CK-MB, Trop I, BNP), and clinical severity (NMDAS = Newcastle Mitochondrial Disease Scale).

**Results:**

Among 11 consecutive patients with MELAS syndrome (73% male, mean age 37.5 ± 10.6 years) cardiac manifestations were found in nine (82%). Pathology was mainly detected using MRI (9 of 11, 82%). Six patients showed diffuse late enhancement in the left ventricle, one a left ventricular ejection fraction (LVEF) below 30%, two with a LVEF in the range of 40–50% in the cardiac MRI, and another five patients presenting diastolic dysfunction as defined by echocardiography. Only one patient with late enhancement on MRI also showed a conduction block in the ECG. There was no correlation between the cardiac manifestations and the NMDAS score or heteroplasmy grade.

**Conclusions:**

Cardiac involvement in MELAS syndrome harboring the MTTL1 m.3243A > G mutation mostly entails cardiomyopathy, which was particularly evident in the cardiac MRI. Only one patient (1/11, 9.1%) had conduction defects. Thus, cardiac testing including cardiac MRI, echocardiography and ECG seems to be important for prognosis of MELAS patients.

**Supplementary Information:**

The online version contains supplementary material available at 10.1186/s13023-025-03534-5.

## Introduction

Mitochondrial diseases (MD) are rare and heterogeneous metabolic diseases in which mutations lead to impaired function of the mitochondria. This results in disturbances in the energy metabolism of the cells. There are many different MD syndromes with a whole range of different symptoms, since mitochondria are found in all cells of the human organs. The cause is that mitochondria are found in all cells of the human organs. Over 400 different genetic defects have been identified to date. Mutations of mitochondrial genes (sporadic or maternal inheritance) or mutations of nuclear DNA (mostly autosmal recessive) can occur. Guidelines and everyday clinical practice usually classify the various manifestations according to clinical syndromes, e.g. chronic progressive external opthalmoplegia (CPEO) or mitochondrial encephalomyopathy, lactic acidosis and stroke-like episodes (MELAS) [1–3].

MELAS is one of the maternally inherited mitochondrial syndromes observed most frequently [4]. The m.3243A > G mtDNA mutation is found in ~ 80% of patients with MELAS syndrome [5]. Underlying diagnostic criteria for MELAS are stroke-like episodes before the age of 40, encephalopathy with epileptic seizures, dementia, or both, and lactic acidosis and ragged-red fibers in skeletal muscle, or both [6–8]. These criteria were updated in 2012 and at least two category A and two category B criteria must be fulfilled. Category A is based on clinical symptoms and neuroimaging findings, including headaches, seizures, hemiplegia, cortical blindness, and acute focal lesions involving the brain cortex. Category B is based on laboratory results with increased plasma or CSF lactate, mitochondrial abnormalities on muscle biopsy (ragged-red fibers—RRF), and a MELAS-related pathogenic variant on genetic testing [9]. In addition, cardiac involvement has been reported in various mitochondrial genotypes and phenotypes [10, 11] and also in MTTL1 m.3243A > G carriers [12]. A meta-analysis showed that patients with MELAS syndrome have the highest prevalence of ECG and echocardiographic abnormalities compared to other mitochondrial diseases [13]. Clinical presentation at onset is heterogeneous and cardiac involvement may include cardiomyopathy and/or cardiac conduction defects and can be found in more than 50% of these patients. In MELAS syndrome, hypertrophic remodeling represents an early pattern of cardiomyopathy, which can lead to dilated cardiomyopathy and results in significant heart rhythm disorders in many MELAS patients. However, hypertrophic cardiomyopathy is more common than dilated cardiomyopathy in these patients [11, 13–15]. Additionally, different types of conduction deficits (atrioventricular block and Wolff-Parkinson-White syndrome) or rhythm disturbances (frequent ventricular ectopic patterns, atrial fibrillation, ventricular tachycardia, and sinus arrest) are common [16, 17]. An analysis of cardiac MRI (3 Tesla) findings in 10 m.3243A > G mutation carriers without MELAS syndrome revealed structural and functional cardiac abnormalities in all of them. A relevant proportion of the patients (n = 4) also had other pre-existing conditions that could trigger cardiomyopathy. The end-diastolic volume, end-systolic volume, and ejection fraction were particularly conspicuous [18]. Therefore, an in-depth analysis of the cardiac involvement of patients with MELAS syndrome and the MTTL1 m.3243A > G mutation is necessary. We hypothesize that cardiac involvement of MELAS syndrome is quite common, if all available cardiac diagnostic options are utilised. Therefore, we analyzed cardiac involvement in 11 German adult patients with MELAS syndrome by cardiac MRI, echocardiography and ECG.

## Materials and methods

### Study design and patient population

From 2007 to 2016, 30 patients with MELAS syndrome from all over Germany were identified. Patient recruitment proved to be somewhat difficult since the disease was already severe in some patients at the time of first contact and they were unable to participate in the study or died after first contact. Ultimately, therefore, n = 11 patients were included in the study, all of whom had a proven MTTL1 m.3243A < G-mutation. Some of the other clinical data and methods (neuropsychological deficits) from 10 patients have been published elsewhere [19]. The study participants or their legal guardian were informed in detail about the procedure and content of the study prior to the study and consented to the investigation. The study was carried out with the approval of the Ethics Committee of the Martin Luther University of Halle-Wittenberg (ethical aprovel code: 184/13.06.07/2, 22.08.2007).

### Recruitment of patients and study procedure

All 30 patients with MELAS syndrome and MTTL1 m.3243A < G mutation were undergoing treatment at the Muscle Centre of the Department of Neurology, University Hospital Halle-Saale. They were contacted by post regarding participation. A telephone response was received from 11 patients. After the telephone contact, an inpatient appointment for diagnostics was arranged. During the inpatient appointment (day 1), a neurological examination with recording of the Disease Scale of the Newcastle Mitochondrial Disease Scale (NMDAS) was carried out. A blood sample was then taken to determine the laboratory parameters. ECG, Long-time-ECG and an echocardiogram were performed on day 2. On day 3, a cardiac MRI and neuropsychological tests were performed (these were published elsewhere [19]. All examinations were carried out at the Muscle Centre of the Department of Neurology, University Hospital Halle-Saale.

### Clinical assessment

All patients were clinically investigated from a special neurologist with knowledge in the field of neuromuscular diseases, got a neurological examination and the BMI was measured. All clinical and previous data was collected in a pseudonymised database. The following data were collected: age, gender, clinical involvement (e.g. migraine, stroke like episodes, seizures, lactic acidosis, diabetes mellitus, ragged red fibres, rate of heteroplasmy, myopathy). All clinical results were than scored in the adapted German version (Part I, II and III) of the NMDAS, a semi-quantitative clinical rating scale to measure the severity of mitochondrial diseases and especially in MELAS syndrome [20]. The NMDAS score contains four sections, A: Current Function (1), B: Systemic Impairment (2), C: Current Clinical Assessment (3) and D: Quality of Life (4). The points of the first three sections are added together. The score for quality of life SF-12 (4) was not analysed in our study. The following grading was used to categorise clinical severity: ≤ 10 mild clinical involvement, between 11 and 20 moderate involvement and ≥ 21 severe involvement.

(Suplement 1).

### Laboratory test

All patients were analyzed for creatine kinase (CK), CK-MB, brain natriuretic peptide (BNP), and tropinin I.

### Cardiac test

Echocardiography was performed using ultrasound systems from GE healthcare (Chicago, Illinois, the United States) and according to the standards of our university clinic (according to Buck et al. [21]. A cardiologist carried out the echocardiography-examination and later they were analyzed by a special cardiologist. The parameters for early diastolic mitral annulus velocity (E’), the ratio of the speed of early left ventricular filling (E) to E’, and also the ratio of early atrial left ventricular filling E/A were taken as the definition of diastolic dysfunction according to Erbel et al. [22]. ECG and laboratory analyses were performed according to local standards and later they were analyzed by a special cardiologist. All data were presented in supplement 2.

*MRI* examinations were performed using a 1.5-Tesla MR machine (Magnetom Sonata Vision, Siemens, Erlangen, Germany). The cardiac MRI work-up included a late enhancement true fast imaging sequence with a phase-sensitive inversion recovery (PSIR) sequence. (usually, an additional FLASH sequence was created). Contrast agent was administered intravenously (0.2 mmol/ kg body weight) with a delay of at least 10 min. Furthermore, a T2-weighted TIRM was acquired to detect edema. However, in less than half of the cases, it was impossible to evaluate the T2-weighted TIRM images adequately because of poor image quality mainly caused by motion artifacts. In addition, cine sequences were carried out in long and short axes as well as in the 4-chamber view. The examination was performed and analysed by a specialist in radiology and cardiac MRI. All data were presented in Supplement 3.

### Statistical methods

Continuous variables were reported as mean ± standard deviation (SD), skewed variables as median and interquartile range (IQR) 25/75, and categorical variables as percentage. All calculations were carried out with SPSS software (V22, SPSS Inc., Chicago, Illinois, USA).

## Results

### Clinical data

Of the 11 patients, eight were male (73%) and three were female (27%). The median age was 44 years, mean age 37.5 ± 10.6 years (range 28–62 years). The mean BMI was 21.6 ± 3.2 kg/m^2^, and mean body surface area was 1.65 ± 0.15 m^2^. Clinically, all patients reported stroke-like episodes and lactic acidosis. Seizures were reported in six of 11 (55%) patients, diabetes mellitus in six of 11 patients (55%), and myopathy in eight of 11 patients (73%). Ragged red fibers were detected in 10 of 11 patients (91%). Median age at earliest symptom was 7 years (range 2–18 years). In eight of 11 patients (73%), a migraine-like headache was present. As previously presented, the NMDAS sscore (part I-III) were high in these subjects (median 35 points, range 9–44; Table 1). The NMDAS revealed mild involvement in 4% (1/11 patients), moderate involvement in 4% (1/11 patients) and severe involvement in 82% (9/11 patients). In six of 11 (55%) patients a family history of mitochondrial disease could be found. In blood samples, the heteroplasmy grade could be analyzed in all patients (median 21, range 9–35), urine samples only in five of 11 (45%) (median 81, range 60–90) and in muscle tissue in two of 11 (18%) (median 48.5, range 32–65).

**Table 1:** Clinical data of patients with MELAS syndrome

Patient	Age	Sex	Migraine	SLE	Seizures	Lactic acidosis	Diabetes	RRF	Myopathy	NMDAS
1	53	F	X	X	–	X	X	X	X	35
2	31	M	X	X	–	X	–	X	–	13
3	44	M	X	X	X	X	–	X	–	36
4	52	F	X	X	–	X	X	–	–	10
5	24	M	X	X	X	X	–	X	X	34
6	28	F	–	X	X	X	–	X	X	29
7	35	M	X	X	X	X	–	X	X	41
8	53	M	–	X	X	X	X	X	X	44
9	62	M	X	X	–	X	X	X	X	42
10	56	M	X	X	X	X	X	X	X	36
11	44	M	–	X	–	X	X	X	X	40

M: male; F: female, SLE: stroke like episodes, X: present, RRF: ragged red fibers.

### Electrocardiogram (ECG)

ECG was performed in all patients. Surprisingly, right branch bundle block was only found in one (1/11, 9.1%). All patients were in sinus rhythm; mean heart rate was 73.3 ± 10.5 beats per minute.

### Laboratory test

Median CK was 2.56 µkat/l (< 3.2 µkat/l), mean CK (3.16 ± 1.62 µkat/l). CK-MB (0.43 ± 0.50 µkat/l) and troponin I (7.9 ± 19.3ng/ml) were in the normal range, whereas mean BNP was slightly elevated (191.2 ± 289pg/ml), > 35pg/ml as a criterion in the definition of heart failure [23]. In three patients the BNP was > 100pg/ml, in one patient above 300pg/ml, and in one patient above 1000pg/ml (Table 2).

**Table 2:** Laboratory test and Heteroplasmy of patients with MELAS syndrome

Patient	CK µkat/l	CK-MB µkat/l	TNT ng/ml	BNP ng/l	BMI kg/m2	Muscle in %	Blood in %	Urine in %
1	4.42	-	0.01	–	20.8	–	23	–
2	2.01	0.11	0.02	6	23.9	–	29	–
3	3.53	0.12	0.04	125	24.2	65	13	81
4	1.91	–	–	26	24.2	–	12	78
5	1.91	1.60	0,03	13	15.8	–	35	85
6	2.19	0.17	–	325	20.1	–	19	–
7	6.83		0.03	199	19.4	–	22	90
8	5.49	0.27	0.07	142	24.2	32	9	–
9	2.62	0.15	–	15	26.8	–	8	60
10	2.56	–	–	53	17.7	–	21	–
11	1.48	0.6	0.05	1008	20.8	–	20	–

### Cardiac MRI (cMRI) and echocardiography

Data are presented in Tables 3 (Cardiac MRI) and 4 (echocardiography). Whereas mean LVEF was in the normal range of 55–70% (54.5 ± 13.8%), one patient had a LVEF below 30% (HFrEF = heart failure with reduced ejection fraction) and two in the range of 40–50% (HFmrEF = heart failure with mid-range ejection fraction). In these three patients, as expected, left ventricular end-diastolic volume (LVDD) was increased (181, 225 and 226ml), whereas mean LVDD was 135.2 ± 49.0ml in the whole group. Moreover, late enhancement as a further sign of cardiac pathology was found in six of 11 patients (54%; Table 4).

**Table 3:** Results of cardiac MRI

Parameter	n = 11
Left ventricular ejection fraction, %	54.5 ± 13.8
Left ventricular enddiastolic volume, ml	135.2 ± 49.0
Left ventricular endsystolic volume, ml	66.3 ± 45.9
Left ventricular systolic volume, ml	68.7 ± 9.4
Myocardial mass, g	152.7 ± 39.5
Right ventricular ejection fraction, %	58.8 ± 13.0
Right ventriculare enddiastolic volume, ml	127.8 ± 38.8
Right ventricular endsystolic volume, ml	57.0 ± 38.4
Right ventricular systolic volume, ml	71.1 ± 7.4
Late enhancement, n (%)	6 (54)

**Table 4:** Results of echocardiography

Parameter	
Left ventricular ejection fraction, %	59.3 ± 12.7
E/A ratio	1.64 ± 0.86
Diastolic dysfunction, n (%)	8 (73)
Diastolic dysfunction, stage, n (%) 0 I II III	3 (28) 4 (36) 2 (18) 2 (18)

On echocardiography, mean LVEF was 59.3 ± 12.7%, as expected higher than LVEF measured by cMRI. Diastolic dysfunction, which can be reliably measured by echocardiography, was found in eight of 11 patients (73%; Table 2).

In summary, systolic or diastolic dysfunction of the left ventricle was found in nine of 11 patients (73% of the entire group) by cMRI or echocardiography.

## Discussion

In the current prospective study, cardiac involvement was found in nine of 11 MELAS patients (82%) with the MTTL1 m.3243A > G mutation. One patient had a LVEF below 30%, which fulfills the criterion of HFrEF, and two patients were in the range of 40–50% (HFmrEF). In another five patients, diastolic dysfunction with preserved ejection fraction (> 50%, HFpEF) was found. Conduction defects were found only in one patient (9.1%). It is interesting to note that the heteroplasmy rate in our group does not appear to have any influence on cardiac involvement. No correlations were found with regard to the relationship between CK-MB or gender and cardiac involvement.

**Fig. 1:**
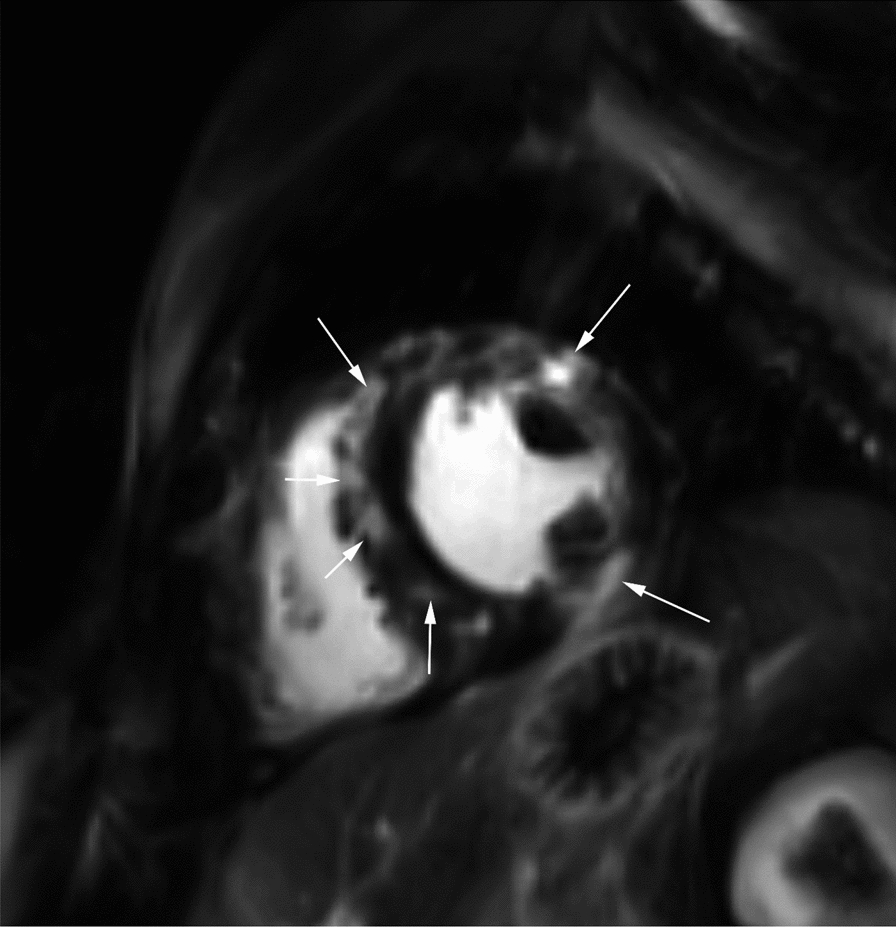
45 year-old male MELAS patient with severe cardiac involvement. MRI PSIR (*Phase-sensitive inversion recovery) *image in short axis orientation 10 min after CE-adminstration. The white arrows mark some of the late-enhancement areas in the ventricular septum and in the inferior and anterior wall of the left ventricle

Earlier studies showed manifest cardiomyopathy or congestive heart failure in nine of 51 (18%) MELAS patients, though without cardiac MRI having been performed. Conduction defects were described in six of 43 (14%) and in four of 30 (13%) of another group of MELAS patients [5]. Yilmaz et al. found in their investigations of three patients with MELAS syndrome using cardiac MRI (1.5 Tesla) edema on T2-weighted images in one patient and late gadolinium enhancement on T1-weighted images in two patients [24]. Late enhancement was found in 54% of the patients (six of 11 patients) in our cohort as a further sign of cardiac pathology (Fig. 1). Later, Yilmaz et al. described a group of seven MELAS patients and four MELAS-like patients (they did not fulfilled the MELAS criteria by Hirano and also the adopted criteria as presented above) without a genetic cause of the disease. In these patients, 10 of 11 (91%) presented at least one abnormal findings in cardiac MRI, among them 73% with a late enhancement [25]. Brambilla et al. investigated 21 patients with the MTTL1 m.3243A > G mutation, but some of them were younger than 18 years and not all criteria of MELAS were fulfilled in these patients. Cardiac MRI was not performed. This was probably why cardiac involvement was found in only 1/3 of the patients [11]. Hendrix et al. analyzed 19 patients with MELAS syndrome and found a clinically relevant echocardiographic abnormality in 47.4% and ECG abnormality in 47.4%. The median NMDAS score study of Hendrix et al. was 22, but in our study the median NMDAS was 35 points, range 9–44 points and 35.9% of our patients were categorised as clinically severely affected. Hendrix et al. found a significantly higher prevalence of TTE abnormalities with severe NMDAS scores. This may explain why we were able to detect a relevant higher impairment on echocardiography in 73% (8/11) of our patients. In contrast to them we found ECG abnormality in just 9.1%. Cardiac MRI was not performed by this group and they and the patients who were included in the MELAS group had only stroke-like episodes in their medical history and therefore did not fulfil the MELAS criteria. [9, 26].

It is important to identify patients with HFpEF and HFmrEF as these patients have a worse prognosis due to heart failure, independent of their symptoms. Thus, patients with MELAS syndrome must not only be treated by neurologists but also by cardiologists to detect cardiac involvement in order to initiate treatment for heart failure, including implantation of an ICD (internal cardioverter defibrillator) for preventing sudden cardiac death, if necessary, as early as possible. We hypothesize that such an algorithm can prevent life-threatening heart rhythm disorders and prolong life in MELAS patients. In addition, cardiac MRI is necessary in patients with MELAS syndrome in order to detect late enhancement.

## Limitations

The small number of patients is due to the rarity of the disease and should be taken into account when making statements about statistical correlations. Compared to other studies, our patients have a higher average age. The lower degree of heteroplasmy can most likely be attributed to the fact that in younger patients with a higher degree of heteroplasmy the risk of death is higher [27]. The rate of heteroplasmy was not available for all patients in all tissues.

## Conclusion

- Cardiac manifestations were found in nine of 11 (82%) MELAS patients with the MTTL1 m.3243A > G mutation.
- It is important to identify patients with HFpEF and HFmrEF as these patients have a worse prognosis.
- Cardiac MRI is necessary to detect late enhancement.

## Supplementary Information

Additional file 1.

Additional file 2.

Additional file 3.

Additional file 4.

## Data Availability

See the supporting data on attached files 1–4.
